# Appetite, oral health and weight loss in community-dwelling older men: an observational study from the Concord Health and Ageing in Men Project (CHAMP)

**DOI:** 10.1186/s12877-021-02169-y

**Published:** 2021-04-16

**Authors:** Sachiko Takehara, Vasant Hirani, F. A. Clive Wright, Vasi Naganathan, Fiona M. Blyth, David G. Le Couteur, Louise M. Waite, Markus J. Seibel, David J. Handelsman, Robert G. Cumming

**Affiliations:** 1 Centre for Education and Research on Ageing, Concord Clinical School, The University of Sydney, and the Ageing and Alzheimer’s Institute, Concord Repatriation General Hospital, Sydney Local Health District, Concord, NSW Australia; 2 Department of Public Health, Tokyo Women’s Medical University, Tokyo, Japan; 3 School of Life and Environmental Sciences, Charles Perkins Centre, The University of Sydney, Sydney, NSW Australia; 4 Concord Clinical School, Concord Repatriation General Hospital, The University of Sydney, Concord, NSW Australia; 5 Department of Geriatric Medicine and Rehabilitation Medicine, Concord Repatriation General Hospital, Sydney Local Health District, Concord, NSW Australia; 6 School of Public Health, Sydney Medical School, The University of Sydney, Sydney, NSW Australia; 7 ANZAC Research Institute, The University of Sydney, Concord Hospital, Sydney, NSW Australia

**Keywords:** Weight loss, Oral health, Tooth loss, Older men, Appetite

## Abstract

**Background:**

Unintended weight loss and the reduction in appetite are common phenomenon among older people. Reduced appetite has been linked to medication related reductions in saliva production, reduced taste ability and poor oral health. Poor appetite can result in reduced nutrient intake ensuing weight loss. It is possible that poor appetite is a mediating step on the causal pathway between oral health and weight loss. This study investigates whether poor oral health and loss of appetite are related to weight loss.

**Methods:**

This is an observational study where data were obtained from the Concord Health and Ageing in Men Project (CHAMP). Information on socio-demographics, appetite and health related behavior was collected by self-completed questionnaire. Intraoral assessment was conducted by calibrated oral health therapists. Height and weight were measured by trained staff. Regression analysis investigated associations between oral health and appetite as risk factors for weight loss.

**Results:**

Participants included 542 community dwelling older males. 99 older men (18.3%) experienced 5% or more weight loss over 3 years. Men who lost weight from baseline had lower BMI and lower body weight, had higher prevalence of frailty and depression, reported poorer appetite, and had fewer teeth (13.8 ± 9.5) than those who did not lose weight (16.3 ± 9.3). Before adjustment, the prevalence ratio (PR) for weight loss was 1.76 (95% Confidence Interval (CI), 1.19–2.59) for participants with 0–19 natural teeth present compared to those with 20 or more teeth. When adding appetite and other variables to the model, the PR for number of teeth and weight loss was unchanged: 1.78 (95% CI, 1.06–3.00). The mediation analysis showed that the indirect effect of appetite on the association between number of natural teeth on weight loss was not found to be significant.

**Conclusion:**

This study found that number of natural teeth present and appetite are independently related to weight change among elderly men in Australia. Tooth loss can increase the risk of swallowing difficulty leading to change in food preference, avoidance of foods and a decrease in energy intake. Our study showed the importance of oral health interventions to encourage maintenance of 20 or more natural teeth in older people.

## Background

Among older people, unintended weight loss is a common phenomenon caused by various pathophysiological, socioeconomic and mental health factors. Past observational studies have suggested weight change is a predictor of outcomes such as premature death and disability among older people. Weight loss of 3 to 5% within 2 to 3 years is associated with a higher risk of mortality in older people [1, 2]. One of the factors associated with weight loss is poor oral health, as suggested by recent cross-sectional and cohort studies [3–5].

Reduced appetite in older people has been linked to medication related reductions in saliva production, reduced taste ability and poor oral health [6]. Poor appetite, often termed the anorexia of ageing, can result in reduced food and nutrient intake, changes in food choice, and ensuing weight loss [6, 7]. It is possible that poor appetite is a mediating step on the causal pathway between oral health and weight loss. Several studies have found that oral health is associated with body weight [3, 4, 8, 9]. However, to our knowledge, there have been no studies that have investigated both poor oral health and poor appetite with regards to weight loss among an older population. The aim of this study was to investigate whether poor oral health and loss of appetite are related to weight loss in community dwelling older males in Australia.

## Methods

### Study participants

**Fig. 1:**
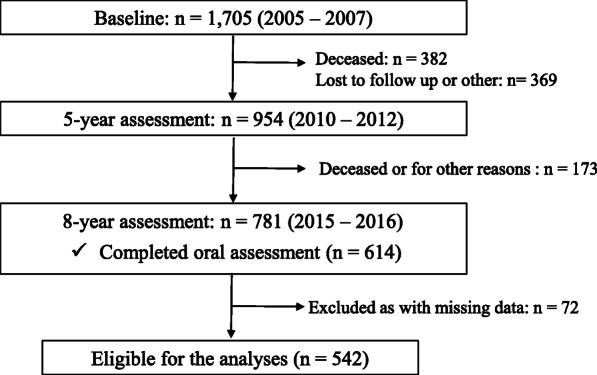
Flowchart of the study

The current observational study was performed with data from the 5 (2012–2013) and 8 (2015–2016) year assessments of the Concord Health and Ageing in Men Project (CHAMP), a longitudinal epidemiologic study of older men in New South Wales (NSW), Australia (Fig. 1). The baseline study was conducted from 2005 to 2007, with male participants aged 70 years and over, living in a defined geographical region (Local Government Areas of Burwood, Canada Bay and Strathfield) [10]. The sampling frame was the NSW Electoral Roll, where registration is compulsory for all citizens of Australia for a representative population sample. The study design has been reported in detail elsewhere [10]. All the eligible men in the study area were invited to join the study excluding men living in residential aged care facilities. In 5th and 8th -year assessments, all older males who participated in the previous assessments were invited to participate in this assessment. All participants were given a verbal explanation of study purpose and methods involved and signed consent forms.

### Anthropometric data

Height and weight were evaluated twice: first at the 5-year follow-up (2010–2012); and then at the 8-year (2015–2016) follow-up study. Height was measured using the Harpenden Stadiometer (Dyfed, UK). Weight was measured wearing light indoor clothing and without footwear on a digital weight scale (Tanita BWB-600). The same scale was used for all measures to maintain measurement fidelity. Body mass index (BMI) was calculated dividing weight (kg) by the square of height (m) [11].

### Weight loss

Weight change of participants over the 3-year period was evaluated by comparing current weight at the 8-year follow-up assessments to weight at the 5-year follow-up assessments: [(weight) – (weight 3-years before)] / (weight) × 100. We defined significant weight loss as a loss of 5% or more over a 3-year period [2, 12]. Participants were dichotomized into two groups: (1) those who had a weight loss of 5% or more over the previous 3-years; and (2) those who had stable weight (either less than 5% weight gain or less than 5% weight loss), or had gained weight of 5% or more in the previous 3-years.

### Oral health assessment

Data regarding oral health was obtained from the 8-year assessment of CHAMP study when the dental study was conducted for the first time.

#### Dentition and dentures

Standard intra-oral assessments were conducted by two calibrated oral health therapists using protocols consistent with Australian dental data collection standards [13]. Calibration procedure and examiners’ reliability were reported previously [13]. Tooth loss, replacement teeth and number of teeth present including 3rd molars were recorded from each participant. Functional Tooth Units (FTUs) were calculated as described by Kayser and Ueno et al. [14, 15]. The maximum number of FTUs is 12 units. FTUs measure the contact of opposing posterior teeth and are therefore used as an estimate of masticatory efficiency [14, 15]. The number of natural teeth present was categorized as either (a) 0 to 19,or (b) 20 or more [14]. Denture wearing status was classed into three categories: (1) full dentures for both mandibular and maxilla, (2) full denture for either mandibular or maxilla, and/or partial denture, and (3) no denture.

#### Mouth dryness

Resting saliva production was estimated by the visual screening method described by Walsh [16]. Calibrated oral health therapists gently rolled subjects’ lower lip out and down, then dried an area on the inside surface of the lip with a gauze, and then observed the formation of droplets of saliva on the lip over a 60 s period. The results were categorized into three groups based on saliva droplets formation: (1) droplet formation within 30 s, (2) droplet formation between 30 and 60 s, or (3) no droplet formation after 60 s. Categories 2 and 3 were classified as ‘dry mouth’ for the analyses.

#### Chewing capacity and oral health variables

Chewing capacity was evaluated by asking participants whether they were able to chew each of the 11 items on a list of foods ranging from ‘soft’ through ‘hard’ texture. Details of the questionnaires and assessment processes have been reported in previous studies [10, 13, 17]. Chewing assessment was based on methods described by Leake, but modified for the ethnic characteristics of the CHAMP study population, which included a high proportion of Italian and Greek migrants [13, 18]. Answers to each food item were dichotomized as ‘yes’ or ‘no/not applicable’. Total responses were later dichotomized as “no problem” in chewing if participants answered yes (able to chew) to over 10 food items or as “limited” chewing capacity if they answered yes to fewer than 10 food items.

Information on self-evaluated oral health and difficulties in swallowing certain foods were also obtained through a self-completed questionnaire, and then dichotomized as either: (a) self-rated oral health (excellent/very good/good vs. fair/poor), and (b) swallowing difficulty (never/hardly ever vs. occasionally/fairly often/very often).

Dietary characteristics (that included both appetite and having a special diet such as low fat diet, diabetic diet, and lactose diet advised by a health professional) were evaluated professionally by the study dietitian. Data were dichotomized as either having a special diet advised by a health professional or not (‘yes’ vs. ‘no’). Appetite was assessed by applying the question: how would you describe your appetite? The response options were: ‘very good’, ‘good’, ‘average’, ‘poor’, and ‘very poor’. The responses were then categorized into two groups: ‘very good, good’ vs. ‘average, poor, very poor. Poor appetite was defined as ‘average’, ‘poor’, and ‘very poor’. This categorization was based on a previous study by van der Meij et al [19].

### Covariates

Sociodemographic characteristics (age, country of birth, income, marital status, post-school education), behavioral (drinking status, smoking status, and physical activity) were collected through self-completed questionnaires. Country of birth was categorized into three groups: Australia; Greece or Italy; and other countries. An income-based assessment was used to characterize income levels: the lowest income category was categorized as ‘all income from aged pension only’; the middle category was income received from ‘age pension plus other income’; and the highest category was ‘income from any possible combinations of superannuation, private income, own business/farm/partnership, wage or salary income, repatriation or veteran’s pension, or other income’ (but ineligible for any age pension due to high income). Marital status was categorized as ‘married or having a partner’, ‘widowed’, ‘divorced or separated’, and ‘never married’. Post school qualification was categorized as having a post-secondary school qualification or not. Smoking status was classified into two categories: ‘never smoked’ or ‘current/previous smoker’.

Comorbidities were assessed using a standardized questionnaire, in which participants indicated they had been told by a physician whether they had one or more of the following health conditions: diabetes, thyroid dysfunction, osteoporosis, Paget’s disease, stroke, Parkinson’s disease, kidney stones, dementia, depression, epilepsy, hypertension, heart attack, myocardial infarction, angina, congestive heart failure, intermittent claudication, chronic obstructive lung disease, liver disease, chronic kidney disease, cancer, osteoarthritis or gout [10]. The number of comorbidities were categorized into either ‘0 - 3’ or ‘≥ 4’ comorbidities.

Physical activity was evaluated by asking ‘Do you take walks for exercise, daily or almost every day?’ The answers were dichotomized as ‘yes, (daily or almost every day)’ or ‘no’.

Depressive symptoms were measured using the short, 15-item version of the Geriatric Depressive Scale (GDS) [20, 21]. A score of five or more points was used as the cut point for clinically important depressive symptoms [22]. Frailty was assessed by trained staff using the five criteria of weight loss/shrinking, weakness, exhaustion, slowness and low activity, following the definition by Fried et al. slightly modified for CHAMP [23, 24]. CHAMP men were categorized as ‘frail’ if they had three or more frailty criteria, ‘pre-frail’ with one or two criteria and ‘robust’ (not frail) without any criteria.

### Ethics statement

The study was performed in accordance with World Medical Association Declaration of Helsinki, and the study protocol and consent procedures were approved by Sydney Local Health District Human Ethics Research Committee (Approval No. HERC/14/CRGH/17).

### Statistical analysis

Chi-squared tests were utilized to examine the bivariate associations between weight loss (weight loss of 5% or more vs. weight loss less than 5%, or weight gain) and categorical variables such as country of birth, income, marital status, post-school qualification, drinking status, smoking status, comorbidities, having a special diet advised by a health professional, appetite, physical activity, frailty and depression. Mean values of age, BMI and body weight (at the time of study and 3-years before), total-FTU and number of teeth present by weight loss category were analyzed using Student’s t-test. Chi-squared tests were performed to examine the relationship between weight loss and oral health categorical variables and between appetite and oral health variables (number of present teeth, FTU, chewing capacity, swallowing difficulty, mouth dryness, self-evaluated oral health).

Poisson models with robust variance estimation were used to examine the association between number of natural teeth and weight loss. Model 1 only included number of teeth and weight loss. Model 2 was adjusted for appetite. In the model 3, age (as a continuous variable), income, comorbidities, country of birth, post school qualification, BMI 3-years previously (as a continuous variable), smoking status, having a special diet and denture wearing status were further entered. Prevalence ratios (PR) and their 95% confidence intervals (CI) were calculated for these models. Calculating variance inflation factors in each Poisson model allowed the detection of any presence of multicollinearity [25]. In order to examine if appetite mediated the relationship between number of teeth present and weight loss, the bootstrapping method was employed using PROCESS macro for SPSS (version 3.5) [26]. The number of bootstrap samples was set to 5000. A two-sided *p*-value < 0.05 was used as the level of statistical significance. Statistical analyses were performed using Statistical Package for Social Sciences (SPSS) for Windows, version 19.0.0 (SPSS, Inc., Chicago IL).

## Results

There were 781 participants who competed the self-completed questionnaire and 614 men who completed the oral health assessment in the 8-year study. After excluding participants with any of missing values, data were available for 542 CHAMP men who completed the oral health assessment, dietary assessment and body measurement. The mean age (SD) of participants at the 5-year of the study was 80.3 years (4.1). The mean BMI (SD) of the men at that time was 27.9 (4.2) and 27.8 (4.3) at 5-year of the study. There were 99 older men (18.3%) who experienced 5% or more weight loss over the preceding 3 years, with 389 older men (71.7%) who maintained a relatively stable weight with either less than 5% weight gain or a weight loss of less than 5%. The remaining 54 older men (10.0%) had 5% or more weight gain. Table 1 (a), (b) shows the characteristics of study participants at the 5 and 8-year assessments by weight loss. As compared to men with stable weight or weight gain of 5% or more, the men who lost weight 5% or more had lower BMI and body weight, higher prevalence of frailty and depression, and were more likely to report poor appetite at the 8-year assessment.

**Table 1:** Distribution of demographic and health conditions of participants by weight loss

a. Average BMI, body weight and age (mean ± SD)							
	Variables	Total population(*N* = 542)	Stable or Weight gain≥5% ^a^ (*N* = 443)		Weight loss ≥5% ^b^(*N* = 99)		*p* value
**5th year assessment (as baseline data)**	Age	80.3 ± 4.1	80.2 ± 4.0		81.5 ± 4.2		0.08
	BMI	27.8 ± 4.3	27.8 ± 4.2		28.1 ± 4.6		0.48
	Body weight	79.5 ± 13.2	79.4 ± 12.9		80.0 ± 14.3		0.68
**8th year assessment**	BMI	27.9 ± 4.2	28.2 ± 4.0		26.3 ± 4.5		< 0.001
	Body weight	78.7 ± 13.5	79.9 ± 13.1		73.2 ± 13.6		< 0.001
b. Demographic, comorbidities, and healthrelated characteristics at 8th year assessments							
Variables	Total population(*N* = 542)		Stable or Weight gain≥5%^a^ (*N* = 443)		Weight loss ≥5% ^b^(*N* = 99)		*p* value
	n	(%)	n	(%)	n	(%)	
**Demographic and health behaviours**							
**Country of Birth:**							
Australia	286	(52.8)	239	(54.0)	47	(47.5)	
Greece/Italy	123	(22.7)	102	(23.0)	21	(21.2)	0.22
Other	133	(24.5)	102	(23.0)	31	(31.3)	
Post school education (No education)	208	(38.4)	171	(38.6)	37	(37.4)	0.47
**Income:**							
Pension only	208	(38.4)	164	(37.0)	44	(44.4)	
Pensions + other	128	(23.6)	106	(23.9)	22	(22.2)	0.38
Other income	206	(38.0)	173	(39.1)	33	(33.3)	
**Marital status:**							
Married/Partner	395	(72.9)	326	(73.6)	69	(69.7)	0.56
Widowed	97	(17.9)	75	(16.9)	22	(22.2)	
Divorced/separated	18	(3.3)	16	(3.6)	2	(2.0)	
Never married	32	(5.9)	26	(5.9)	6	(6.1)	
**Drinking status:**							
Life-long abstainer	45	(8.3)	33	(7.4)	12	(12.1)	
Ex-drinker	76	(14.0)	56	(12.6)	20	(20.2)	
Safe-drinker	383	(70.7)	323	(72.9)	60	(60.6)	0.07
Unsafe-drinker	38	(7.0)	31	(7.0)	7	(7.1)	
Smoking status (Current or previous smoker)	323	(59.6)	260	(58.7)	63	(63.6)	0.43
**Comorbidities**							
Comorbidities ≥4	155	(28.6)	121	(27.3)	34	(34.3)	0.18
Arthritis	283	(52.2)	234	(52.8)	49	(49.5)	0.58
Diabetes mellitus	117	(21.6)	87	(19.6)	30	(30.3)	0.03
Heart attack	108	(19.9)	86	(19.4)	22	(22.2)	0.53
Stroke	46	(8.5)	34	(7.7)	12	(12.1)	0.16
Hypertension	299	(55.2)	245	(55.3)	54	(54.5)	0.91
Cancer	104	(19.2)	84	(19.0)	20	(20.2)	0.43
**Health related characteristics**							
Take walks daily/almost everyday	285	(52.6)	236	(53.3)	49	(49.5)	0.50
**Frailty:**							
robust	137	(25.3)	124	(28.0)	13	(13.1)	
pre-frail	311	(57.4)	261	(58.9)	50	(50.5)	< 0.001
frail	94	(17.3)	58	(13.1)	36	(36.4)	
Depression (Geriatric Depression Scale ≥5)	90	(16.6)	66	(14.9)	24	(24.2)	0.04
Special diet advised by a health professional	59	(10.9)	48	(10.8)	11	(11.1)	0.94
**Appetite:** ^**c**^							
Very good	107	(19.7)	97	(21.9)	10	(10.1)	
Good	233	(43.0)	193	(43.6)	40	(40.4)	
Average	172	(31.7)	133	(30.0)	39	(39.4)	0.01
Poor	24	(4.4)	16	(3.6)	8	(8.1)	
Very poor	6	(1.1)	4	(0.9)	2	(2.0)	

^a^ Stable weight with either less than 5% weight gain or less than 5% weight loss, or had gained weight 5% or more in the previous 3-years between 5-year and 8-year assessments

^b^ Weight loss of 5% or more over 3-years between 5-year and 8-year assessments

^c^ Appetite was assessed by applying the question: how would you describe your appetite? The response options were: 'very good', 'good', 'average', 'poor', or 'very poor'

The mean number of natural teeth present was 16.3 ± 9.3 among men with stable weight or 5% or more weight gain. This was statistically significantly higher than among those who lost weight by 5% or more (13.8 ± 9.5, *p* = 0.02). Table 2 presents data on oral health status by weight loss category. 52.6% of men with stable weight or 5% or more weight gain had 0–19 natural teeth, compared to 68.7% of men who lost 5% or more weight.

**Table 2:** Oral health status by weight loss

Variables	Total population (*N* = 542)		Stable or Weight gain ≥5%^a^ (*N* = 443)		Weight loss ≥5% ^b^ (*N* = 99)		
	n	(%)	n	(%)	n	(%)	*p* value
Has < 20 natural teeth	301	(55.5)	233	(52.6)	68	(68.7)	< 0.01
Denture wearing status							
Full denture (mandibular+maxillary)	71	(13.1)	55	(12.4)	16	(16.2)	
Single full denture and/or partial denture	238	(43.9)	190	(42.9)	48	(48.5)	0.21
No denture	233	(43.0)	198	(44.7)	35	(35.4)	
Limited chewing capacity ^c^	152	(28.0)	119	(26.9)	33	(33.3)	0.22
Swallowing difficulty (Occasionally/Fairly often/Very often)	65	(12.0)	46	(10.4)	19	(19.2)	0.03
Mouth dryness (Dry: no droplet within 30 s)	346	(63.8)	280	(63.2)	66	(66.7)	0.56
Self-evaluated oral health (Fair/Poor)	151	(27.9)	121	(27.3)	30	(30.3)	0.54

^a^ Stable weight with either less than 5% weight gain or less than 5% weight loss, or had gained weight 5% or more in the previous 3-years between 5-year and 8-year assessments

^b^ Weight loss of 5% or more over 3-years between 5-year and 8-year assessments

^c^ Chewing capacity were evaluated by asking “Are you currently able to chew?” for 11 food items. Subjects who answered "yes" to 10 - 11 food items were classified as "no problem", or otherwise classified as "limited"

Characteristics of oral health by appetite are presented in Table 3. Among the study participants, 340 people (62.7%) reported their appetite as ‘Very good’ or ‘Good’. There were no statistically significant differences in total FTUs or number of natural teeth present by appetite. However, participants who reported appetite as ‘Average’, ‘Poor’, or ‘Very poor’ were more likely to wear dentures, have limited chewing capacity, mouth dryness, and have lower self-evaluated oral health.

**Table 3:** Oral health status by appetite^a^

Variables	Total population (*N* = 542)		Good appetite^b^ (*N* = 340)		Poor appetite^b^ (*N* = 202)		
			n	%	n	%	*p* value
Number of natural teeth (mean ± S.D.)	15.8 ± 9.3		16.1 ± 9.4		15.4 ± 9.2		0.39
Total FTU (mean ± S.D.)	7.7 ± 3.7		7.7 ± 3.8		7.7 ± 3.6		0.98
Has < 20 natural teeth	301	(55.5)	181	(53.2)	120	(59.4)	0.162
Denture wearing status							
Full denture (mandibular + maxillary)	71	(13.1)	45	(13.2)	26	(12.9)	
Single full denture and/or partial denture	238	(43.9)	134	(39.4)	104	(51.5)	0.02
No denture	233	(43.0)	161	(47.4)	72	(35.6)	
Limited chewing capacity ^c^	152	(28.0)	82	(24.1)	70	(34.7)	0.01
Swallowing difficulty (Occasionally/Fairly often/Very often)	65	(12.0)	41	(12.1)	24	(11.9)	0.95
Mouth dryness (Dry: no droplet within 30 s)	346	(63.8)	204	(60.0)	142	(70.3)	0.02
Self-evaluated oral health (Fair/Poor)	151	(27.9)	80	(23.5)	71	(35.1)	< 0.01

^a^ Appetite was assessed by applying the question: how would you describe your appetite? The response options were: 'very good', 'good', 'average', 'poor', or 'very poor'

^b^ Good appetite was defined as very good or good. Poor appetite was defined as average, poor, and very poor

^c^ Chewing capacity were evaluated by asking “Are you currently able to chew?” for 11 food items. The participants who answered "yes" to 10 - 11 food items were classified as "no problem", or otherwise classified as "limited"

Table 4 provides findings from Poisson regression models where weight change (‘5% or more weight loss’ vs. ‘stable weight or weight gain’) was the dependent variable and the variable – number of natural teeth present - was the independent variable. There was a statistically significant association (model 1) between number of teeth and weight loss (PR = 1.76, 95% CI = 1.19–2.59). Similarly, the association remained (PR = 1.73, 95% CI = 1.18–2.55) after being adjusted for appetite (model 2). The addition of appetite (model 2) did not change the strength of association between number of teeth and weight loss. Model 2 was further adjusted by the variables: age (as continuous variable), income, co-morbidities, country of birth, post school qualification, BMI at 3-year interval before (as continuous variable), smoking status, having special diet and denture wearing status (model 3). The association between number of teeth present and weight loss remained significant in this fully adjusted model 3 (PR = 1.78, 95% CI = 1.06–3.00).

**Table 4:** Multivariate poisson regression on weight loss (reference: people with stable weight, or weight gain ≥5% in the previous 3-years)

	PR (95% CI)
Model 1	
Natural teeth present (reference: 20 ~ 32): 0 ~ 19	1.78 (1.06–3.00)
Model 2 (adjusted by appetite)	
Natural teeth present (reference: 20 ~ 32): 0 ~ 19	1.73 (1.18–2.55)
Model 3 (adjusted by multivariables^a^)	
Natural teeth present (reference: 20 ~ 32): 0 ~ 19	1.77 (1.05–2.97)

^a^: It includes following variables: appetite, age, income, comorbidities, country of birth, post school qualification, BMI 3-year before, smoking, special diet and denture wearing status

To investigate whether the number of natural teeth is independently associated with weight loss, a simple mediation analysis was performed. The outcome variable for the analysis was weight loss, the predictor variable was number of natural teeth, and the mediator was appetite. The mediating effect of appetite on weight loss was not found to be significant (effect = − 0.02, 95%CI (− 0.09, 0.05)).

## Discussion

This study provides new epidemiological evidence supporting the association of oral health (represented by number of natural teeth present), appetite and weight loss in community dwelling older men in Australia. A number of previous publications have reported poor appetite as an important determinant of nutritional deficiency and of weight loss among older people [6, 7, 19]. Poor oral health, including wearing dentures, poor dentition, and mouth dryness, are known to be associated with loss of appetite [27]. These previous findings suggest that poor oral health might contribute to weight loss with poor appetite as a mediator. However, our study has shown that poor oral health is an independent risk factor for weight loss in older men.

Poor appetite is frequently observed in association with ageing [28]. It is important to note that nearly 40% of our participants reported poor appetite. This prevalence is consistent with previous studies [28]. We found that men with poorer appetite were more likely to wear dentures, have limited chewing capacity, have mouth dryness, and have lower self-evaluated oral health status. Dormenval et al. also reported that dry mouth is associated with wearing dentures, difficulty in chewing and swallowing, and poorer appetite among hospitalized elderly patients in Switzerland [29]. Kamel et al. examined an adult population in the United Kingdom, and found that dry mouth, or reduced salivary secretion was associated with poorer taste sensitivity and reduced health- related quality of life [30]. Furthermore, Donini et al. examined hospitalized elderly in Italy, reporting that those with poor appetite had limited chewing capacity, swallowing difficulty, and reduced tasted sensitivity [31]. All these reports are consistent with the findings from the present study. However, specific biological mechanisms behind these associations between appetite and oral health are not understood [28].

Only a small number of studies have focused on the association between oral health status and weight loss [3–5]. These previous studies have findings that are consistent with ours [3–5]. However, to our knowledge, no previous studies have considered both number of natural teeth present and appetite in relation to weight loss.

Weyant et al. analyzed data from the Health ABC study, a longitudinal cohort study among a community-dwelling, well-functioning population aged 65 and older in Pennsylvania and found that weight loss was associated with having a compromised oral health status, including lower number of natural teeth present and gingival inflammation [3]. Although the weight of participants was measured twice, oral health examinations were conducted only once. Ritchie et al. conducted a cross-sectional study among community dwelling people aged 70 and older in UK and found that edentate people (having no natural teeth) were more likely to experience weight loss of 4% over a 1 year period [4]. Again, oral health status was assessed by dental professionals only at the baseline, and participants’ weight at the follow-up assessment was self-reported - obtained by telephone interview. A cross sectional study by Nakamura et al. involved data collected exclusively by self-completed questionnaires from 96,794 Japanese men and women aged 65 years over [5]. They found that fewer than 20 natural teeth and poor food intake were both associated with weight loss of 2 ~ 3 kg over a 6-month period [5]. All data in their study were self-reported including weight loss and number of teeth.

The strengths of the present study include the measurement of body weight at two points in time and the objective assessments of oral health, with an interval of 3 years apart. However, it is acknowledged that the final study sample size was relatively small. A further strength of this study is that the CHAMP study has been designed to include a representative group of older community-dwelling Australian men, as shown by the similarity of sociodemographic and health characteristics compared to the nationally representative MATeS study [32]. However, a cautious interpretation is necessary when generalizing our study results to older males throughout Australia as this was an urban population, did not include rural or regional sampling, and other state populations of Australia have a different ethnic diversity and socioeconomic factors.

The main limitation is that the study analyzed cohort data on weight changes over a 3-year period, while the other information, including oral health and appetite, was collected only in year-8 of the study. Thus, the direction of the relationship of weight loss with oral health conditions and appetite cannot be definitely clarified. Malnutrition was solely evaluated by weight loss in our study, not considering BMI. Unintentional weight loss is related to an increased risk of all-causes of mortality in many studies, yet the magnitude of effect is different by levels of BMI [33, 34]. The association of BMI with mortality has been well established [35].

We did not use BMI as a dependent variable as there were only 4 participants who had a BMI ≤ 18.5 at 5th-year assessment. Future research would be more meaningful if both BMI and weight loss were included. A further limitation is that a large number of participants were lost by the follow up assessment. This may have resulted in potential selection biases. The smaller number of participants also reduced the power of statistical tests to detect significant associations between oral health and weight loss. Non-responders and people living at residential aged care facilities tend to be older and more likely to experience unintentional weight loss. It is therefore possible that factors associated with weight loss were not fully accounted for in our analysis. The study may also be influenced by survival bias, where health status of the surviving participants is better compared with those who died or withdrew. A final limitation is that our study only included community living older men, which may limit generalizability to older men living in aged care facilities or older women.

## Conclusions

In conclusion, this study found that the number of natural teeth present and appetite are independently related to weight change among community dwelling elderly men in Australia. Our findings indicate that participants with fewer than 20 natural teeth, and those with poorer appetite are at a higher risk of weight loss. Tooth loss can increase the risk of swallowing difficulty leading to change in food preference, avoidance of foods and a decrease in energy intake. Our study shows the importance of oral health interventions to encourage maintenance of 20 or more natural teeth in older people. Further research using longitudinal data is required to fully elucidate how the number of natural teeth relates to weight loss, and how appetite is related to oral health factors such as swallowing, chewing, and mouth dryness.

## Data Availability

The datasets generated during and/or analyzed during the current study are available from the corresponding author on reasonable request with permission of the CHAMP Management Team.

## Abbreviations

BMI: Body mass index
CHAMP: the Concord Health and Ageing in Men Project
CI: Confidence interval
FTUs: Functional Tooth Units
PR: the prevalence ratio
SD: Standard deviation
